# Cellular and Mathematical Analyses of LUBAC Involvement in T Cell Receptor-Mediated NF-κB Activation Pathway

**DOI:** 10.3389/fimmu.2020.601926

**Published:** 2020-11-23

**Authors:** Daisuke Oikawa, Naoya Hatanaka, Takashi Suzuki, Fuminori Tokunaga

**Affiliations:** ^1^ Department of Pathobiochemistry, Graduate School of Medicine, Osaka City University, Osaka, Japan; ^2^ Division of Mathematical Science, Department of Systems Innovation, Graduate School of Engineering Science, Osaka University, Osaka, Japan; ^3^ Center for Mathematical Modeling and Data Science, Osaka University, Osaka, Japan

**Keywords:** linear ubiquitin, LUBAC, mathematical model, NF-κB, T cell receptor, CBM complex

## Abstract

The LUBAC ubiquitin ligase complex, composed of the HOIP, HOIL-1L, and SHARPIN subunits, stimulates the canonical nuclear factor-κB (NF-κB) activation pathways through its Met1-linked linear ubiquitination activity. Here we performed cellular and mathematical modeling analyses of the LUBAC involvement in the T cell receptor (TCR)-mediated NF-κB activation pathway, using the Jurkat human T cell line. LUBAC is indispensable for TCR-induced NF-κB and T cell activation, and transiently associates with and linearly ubiquitinates the CARMA1-BCL10-MALT1 (CBM) complex, through the catalytic HOIP subunit. In contrast, the linear ubiquitination of NEMO, a substrate of the TNF-α-induced canonical NF-κB activation pathway, was limited during the TCR pathway. Among deubiquitinases, OTULIN, but not CYLD, plays a major role in downregulating LUBAC-mediated TCR signaling. Mathematical modeling indicated that linear ubiquitination of the CBM complex accelerates the activation of IκB kinase (IKK), as compared with the activity induced by linear ubiquitination of NEMO alone. Moreover, simulations of the sequential linear ubiquitination of the CBM complex suggested that the allosteric regulation of linear (de)ubiquitination of CBM subunits is controlled by the ubiquitin-linkage lengths. These results indicated that, unlike the TNF-α-induced NF-κB activation pathway, the TCR-mediated NF-κB activation in T lymphocytes has a characteristic mechanism to induce LUBAC-mediated NF-κB activation.

## Introduction

Nuclear factor-κB (NF-κB) is a pivotal transcription factor controlling innate and acquired immune responses, inflammation, and anti-apoptosis (1, 2). Therefore, impaired NF-κB activity is implicated in diverse diseases, including cancers, metabolic syndrome, and inflammatory, autoimmune, and neurodegenerative diseases. NF-κB activation is typically classified into canonical and non-canonical pathways (1, 2). The canonical NF-κB pathway is activated by stimulations with proinflammatory cytokines, pathogen-associated molecular patterns (PAMPs), and antigen receptors, as well as by genotoxic stress. In the canonical pathway, activation of the IκB kinase (IKK) complex, composed of the IKKα and IKKβ kinases and a regulatory subunit of NEMO, results in the nuclear translocation of NF-κB factors, composed of homo/hetero-dimers of p50, RelA (p65), and c-Rel (1, 2). In contrast, the noncanonical NF-κB pathway functions in different aspects of immune and inflammatory responses through the activation of the IKKα dimer, and predominantly translocates homo/hetero-dimers of p52 and RelB into the nucleus (3).

The ubiquitin system, composed of ubiquitin-activating enzyme (E1), ubiquitin-conjugating enzyme (E2), and ubiquitin ligase (E3), regulates various cellular functions (4, 5). In NF-κB signaling, multiple ubiquitinations, such as Lys(K)63-, K11-, K48-, and Met(M)1-linked ubiquitin chains, reportedly function in the course of IKK activation and degradation of inhibitors of NF-κB, IκBs (6). LUBAC is an E3 complex, comprising the HOIP (also known as RNF31), HOIL-1L (RBCK1), and SHARPIN subunits, that specifically generates the M1-linked linear ubiquitin chain, and activates the canonical NF-κB pathway through the linear polyubiquitinations of NEMO and RIP1 upon stimulation by inflammatory cytokines, such as TNF-α and IL-1β (7–9). Since NEMO contains a linear ubiquitin-specific binding site, the so called UBAN domain, the linear ubiquitin chain functions as a scaffold to recruit and activate the canonical IKK (10, 11). LUBAC and its linear ubiquitination activity participate in several canonical NF-κB pathways induced by proinflammatory cytokines such as PAMPs, T cell receptor (TCR), genotoxic stress, and NOD2-mediated pathways (12, 13), but not in the B cell receptor (BCR)-mediated canonical or the noncanonical NF-κB activation pathways (14, 15).

Importantly, LUBAC binds negative regulators of deubiquitinases (DUBs), such as OTULIN and the CYLD-SPATA2 complex, through the N-terminal PUB domain of HOIP (16–18). OTULIN, an ovarian tumor (OTU)-family DUB, directly binds to HOIP and exclusively cleaves M1-linked ubiquitin chains. OTULIN plays crucial roles in limiting cell death and inflammation (19). In contrast, CYLD is a ubiquitin-specific protease (USP)-family DUB that was initially identified as a cylindromatosis tumor suppressor gene in humans (20). CYLD downregulates the NF-κB activation pathway by hydrolyzing K63- and M1-linked ubiquitin chains (21), and regulates innate immune signaling (22). Importantly, the USP domain of CYLD binds to the PUB domain of SPATA2, and the PUB-interacting motif (PIM) in SPATA2 associates with the PUB domain of HOIP (18, 23–25). Therefore, LUBAC is a unique E3-DUB complex to scrap-and-build linear ubiquitin chains.

TCR recognizes major histocompatibility complex (MHC) molecules expressed on the surface of antigen-presenting cells (26). In the TCR-mediated NF-κB activation pathway, the protein tyrosine kinase ZAP70 is initially activated upon co-stimulation through TCR and CD28 (27), leading to the activation of protein kinase Cθ (PKCθ). PKCθ phosphorylates the scaffold protein CARMA1 (CARD-containing MAGUK protein 1, also called CARD11 and Bimp3) (28, 29). The activated CARMA1 then recruits heterodimers of B cell lymphoma 10 (BCL10) and the paracaspase mucosa-associated lymphoid tissue lymphoma translocation protein1 (MALT1) to form the oligomerized CARMA1-BCL10-MALT1 (CBM) complex, which functions as a scaffold to activate the NF-κB and MAP kinase signaling pathways (28, 29). In TCR signaling, LUBAC linearly ubiquitinates BCL10 (30–32), and MALT1 cleaves HOIL-1L upon stimulation (33, 34). However, the detailed functions of LUBAC in the TCR-mediated NF-κB pathway remain elusive. The stimulation-dependent cytosol-nucleus oscillation of NF-κB and its effects on gene expression have been analyzed mathematically (35–38). However, a mathematical analysis of LUBAC-mediated linear ubiquitination and OTULIN/CYLD-induced deubiquitination in TCR-mediated NF-κB activation has not been performed. Therefore, we have investigated the cellular function and performed a mathematical simulation for the involvement of LUBAC in the TCR-mediated NF-κB activation, using the Jurkat human T cell line.

## Materials and Methods

### Plasmids

The open reading frames of human cDNAs were amplified by reverse transcription-PCR. Mutants of these cDNAs were prepared by the QuikChange method, and the entire nucleotide sequences were verified. The cDNAs were ligated to the appropriate epitope sequences and cloned into the pcDNA3.1 (Invitrogen), pCAGGS (Addgene), and pGEX-6P-1 (Addgene) vectors. For lentiviral transduction, pCSII-CMV-RfA-IRES-Blast (RIKEN BioResource Research Center) was used.

### Reagents

The following reagents and kits were obtained as indicated: recombinant human TNF-α (BioLegend), phorbol 12-myristate 13-acetate (PMA, Sigma-Aldrich), ionomycin (Wako), Human IL-2 Instant ELISA (eBiosciences), and NE-PER Nuclear and Cytoplasmic Extraction Reagent Kit (Pierce).

### Antibodies

The following antibodies were used for immunoblot analyses: P-p105 (#4806; 1:1,000), p105 (#3035; 1:1,000), P-p65 (#3033; 1:1,000), p65 (#8242; 1:1,000), P-IκBα (#9246; 1:1,000), IκBα (#4812; 1:1,000), P-JNK (#4668; 1:1,000), JNK (#9252; 1:1,000), PARP (#9542; 1:1,000), P-ZAP70 (#2701; 1:1,000), ZAP70 (#3165; 1:2,000), P-IKKα/β (#2697; 1:1,000), CARMA1 (#4435; 1:1,000), MALT1 (#2494; 1:1,000), BCL10 (#4237; 1:1,000), CYLD (#8462; 1:1,000), OTULIN (#14127; 1:1,000), and GST (#2622; 1:1,000) were obtained from Cell Signaling. HOIL-1L (sc-393754; 1:250), IKKα/β (sc-7607; 1:1,000), and c-Myc (sc-40; 1:1,000) were purchased from Santa Cruz Biotechnology. HOIP (ab125189, Abcam; 1:1,000), NEMO (ab178872, Abcam; 1:3,000), SHARPIN (14626-1-AP, Proteintech; 1:3,000), tubulin (CLT9002, Cedarlane; 1:3,000), FLAG (clone M2, F1840, Sigma-Aldrich; 1:1,000), DYKDDDDK (1E6; HRP-conjugate) (015-22391, Wako; 1:10,000), Myc (#562, MBL; 1:2,000), linear ubiquitin (clone LUB9, MABS451, Millipore; 1:1,000), and linear ubiquitin (1F11/3F5/Y102L, Genentech; 1:20,000) were also used. For immunoprecipitation, the following antibodies were used: HOIL-1L (sc-49718, Santa Cruz Biotechnology; 3 µg), c-Myc (sc-40, Santa Cruz Biotechnology; 1 µg), FLAG (clone M2, F1840, Sigma-Aldrich; 1 µg), MALT1 (sc-46667, Santa Cruz Biotechnology; 3 µg), CARMA1 (sc-166910, Santa Cruz Biotechnology; 3 µg), BCL10 (sc-5273, Santa Cruz Biotechnology; 3 µg), NEMO (200-401-GM7, ROCKLAND; 3 µg), and normal mouse IgG (sc-2025, Santa Cruz Biotechnology; 3 µg). For cell stimulation, agonistic anti-CD3 (300314, BioLegend) and anti-CD28 antibodies (302914, BioLegend) were used, and the PE anti-human CD69 antibody (310905, BioLegend) was utilized for flow cytometry.

### Cell Culture and Transfection

Jurkat cells (Clontech) were maintained in RPMI 1640 medium, containing 10% FBS, 100 IU/ml penicillin G and 100 μg/ml streptomycin, at 37°C under a 5% CO_2_ atmosphere. HEK293T cells (ATCC) were cultured in DMEM containing 10% fetal bovine serum (FBS) and antibiotics. Transfection experiments were performed using Lipofectamine 2000, PEI (polyethylenimine), or TurboFect (Thermo Fisher). Electroporation of Jurkat cells was performed with a Gene Pulser Xcell Electroporation System (Bio-Rad) at 250 V with 975 µF. For the stable expression of FLAG-tagged OTULIN-WT in *OTULIN*-KO Jurkat cells, lentiviral infection followed by the selection with 5 µg/ml blasticidin was performed.

### Construction of Knockout Cells

The HOIP-deficient *RNF31*-KO Jurkat cells were constructed as described previously (39). The gRNA_cloning vector (#41824) and pCAG-hCas9 (#51142) were obtained from Addgene. The nucleotide sequences 5’- AACAAGAATTGTAATGACCC -3’ in exon 9 of the human *CYLD* gene, and 5’- ATTAAGCGTAGCTCCTGAAA -3’ in exon 3 of the human *OTULIN* gene, were selected as the targets for the gRNA. These plasmids and a puromycin-resistant vector (pXS-Puro) were co-electroporated into Jurkat cells. Two days after transfection, cells were selected with puromycin for 3 weeks, and then cell clones were obtained by limiting dilution. The deficiency of the CYLD or OTULIN protein was confirmed by immunoblotting, and the nucleotide mutations were confirmed by sequencing.

### SDS-PAGE and Immunoblotting

Samples were separated by SDS-PAGE and transferred to PVDF membranes. After blocking in Tris-buffered saline containing 0.1% Tween-20 (TBS-T) with 5% skim-milk or bovine serum albumin (BSA), the membrane was incubated with the appropriate primary antibodies, which are diluted in TBS-T containing 5% w/v BSA, followed by an incubation with horseradish peroxidase-conjugated secondary antibodies (GE Healthcare). The chemiluminescent images were obtained with an LAS4000 imaging analyzer (GE Healthcare) or a Fusion Solo S imaging system (Vilber). Quantification of protein bands was performed with ImageJ software, according to the manufacturer’s instructions.

### Quantitative PCR (qPCR)

Cell lysis, reverse-transcription, and qPCR were performed with SuperPrep Cell Lysis, RT Kit for qPCR, and Power SYBR Green PCR Master Mix (Life Technologies) respectively, according to the manufacturer’s instructions. Quantitative real-time PCR was performed with a Step-One-Plus PCR system (Applied Biosystems) by the ΔΔCT method, using the following oligonucleotides: *NFKBIA* sense, 5’-CGGGCTGAAGAAGGAGCGGC-3’ and *NFKBIA* anti-sense, 5’-ACGAGTCCCCGTCCTCGGTG-3’; *TNFAIP3* sense, 5’-CATGCATGCCACTTCTCAGT-3’, and *TNFAIP3* anti-sense, 5’-CATGGGTGTGTCTGTGGAG-3’; *IL-2* sense, 5’-CTGGAGCATTTACTGCTGGATTT-3’; *IL-2* anti-sense, 5’-TGGTGAGTTTGGGATTCTTGTAATT-3’; *GAPDH* sense, 5’-AGCAACAGGGTGGTGGAC-3’, and *GAPDH* anti-sense, 5’-GTGTGGTGGGGGACTGAG-3’.

### Flow Cytometry

Parental and *HOIP*-deficient Jurkat cells were stimulated with anti-CD3 and anti-CD28 antibodies for 20 h. Afterwards, 2×10^5^ cells were stained with the PE-CD69 antibody for 30 min on ice, washed and analyzed with a FACSVerse flow cytometer, using the FACSuite software (Becton Dickinson).

### Immunoprecipitation and Detection of Linear Ubiquitination

For the detection of linear ubiquitination, Jurkat cells (5×10^7^ cells) were heated at 95°C for 5 min in 1% SDS-containing lysis buffer, which includes 50 mM Tris-HCl (pH 7.4), 1% NP-40, 0.1% sodium deoxycholate, 150 mM NaCl, 1 mM EDTA, 2.5 µM *N*-ethylmaleimide, and a protease inhibitor cocktail (Roche). The samples were then diluted 10-fold with SDS-free lysis buffer. For the protein interaction assay, Jurkat cells (1×10^8^ cells) or HEK293T (5×10^6^ cells) were lysed with NP-40 buffer (20 mM Tris-HCl (pH 7.4), 0.2% NP-40, 150 mM NaCl and protease inhibitor cocktail). Immunoprecipitation was performed with the indicated antibody together with protein G agarose beads (GE Healthcare).

### *In Vitro* Canonical IKK Assay

Parental and *OTULIN*-deficient Jurkat cells (2×10^7^ cells) were stimulated with PMA and ionomycin for the indicated durations. The cells were then lysed in buffer, containing 50 mM Tris-HCl (pH 7.5), 150 mM NaCl, and 1% Triton X-100 (w/v), and immunoprecipitated with an anti-NEMO antibody and Protein A Sepharose (GE Healthcare). After extensive washing, the beads were suspended in buffer containing 50 mM Tris-HCl (pH 7.5) and 5 mM MgCl_2_. The immunoprecipitates were incubated for 30 min at 30 °C with 5 μg/ml of GST-IκBα (aa 1–54), prepared as described (40), in a 20 μl reaction, containing 50 mM Tris-HCl (pH 7.5), 5 mM MgCl_2_, and 1 mM ATP, followed by immunoblotting.

### Statistical Analysis

One-way ANOVA followed by a post-hoc Tukey HSD test was performed, using the GraphPad Prism software. For all tests, a *P* value of less than 0.05 was considered statistically significant.

### Mathematical Simulation

We constructed mathematical models of NF-κB signaling based on the law of mass action, as described previously (38). In order to investigate the effect of the CBM complex on IKK activation in T cells, the following two models were constructed. The first was the CBM simplify model (CBM_SM), which includes the reaction of IKK activation depending on the linear ubiquitin chain of NEMO and the CBM complex. In this model, we simulated the effect of the CBM complex on the IKK activation by changing the parameters related to ubiquitination. The reaction involves the transient binding of LUBAC with NEMO or CBM, followed by the LUBAC-mediated linear ubiquitination of the proteins in the bound state.

L+NEMO → LNEMO, LNEMO → LNEMOu

L+CBM → LCBM, LCBM → LCBMu

After dissociation, the LUBAC-bound NEMO or CBM was postulated to exist in a temporarily inactive form (prime symbol (′) represents inactive state).

LNEMO → L+NEMO′

LCBM → L+CBM′

The linear ubiquitin chains are cleaved by DUBs, such as CYLD and OTULIN. Since CYLD and OTULIN stably bind LUBAC, LUBAC/NEMOu and NEMOu may have different deubiquitination coefficients. The model is characterized by the following equations, and parameters are shown in **Table 1**;

$$\begin{array}{l} \frac{dCBM}{dt}=-k_{C}\;CBM\;\left(t\right)(L_{tot}-LCBM\left(t\right)-LCBM_{u}\left(t\right)\;\\ \;-LIKK\left(t\right)-LIKK_{u}\left(t\right)) \end{array}$$

$$\begin{array}{l} \frac{dLCBM}{dt}=\;k_{C}\;CBM\;\left(t\right)\;(L_{tot}-LCBM\left(t\right)-LCBM_{u}(t)-LIKK\left(t\right)\\ \;-LIKK_{u}\left(t\right))-\left(u_{C}+l_{C}\right)\;LCBM\left(t\right)+d_{C}\;LCBM_{u}\left(t\right)\; \end{array}$$

$$\frac{dLCBM_{u}}{dt}=u_{C}\;LCBM\left(t\right)-\left(d_{C}+l_{C}\right)\;LCBM_{u}\left(t\right)\;$$

$$\frac{dCBM_{du}\;}{dt}=l_{C}\;LCBM_{u}\left(t\right)-a\;d_{C}CBM_{du}\left(t\right)\;$$

$$\begin{array}{l} \frac{dLIK}{dt}=-k_{N}\left(L_{tot}-LCBM\left(t\right)-LCBM_{u}\left(t\right)-LIKK\left(t\right)-LIKK_{u}\left(t\right)\right)\;(IKK_{tot}-IKK_{u}(t)\\ \;\;\;\;\;\;\;\;\;\;\;\;\;\;\;\;\;\;\;\;\;-LIKK(t)-LIKK_{u}(t)-IKK_{p}(t)-IKK_{up}(t)-IKKd(t)-IKKd_{u}(t)\\ \;\;\;\;\;\;\;\;\;\;\;\;\;\;\;\;\;\;\;\;\;-LIKK_{p}(t)-LIKK_{up}(t)-IKKd_{p}(t)-IKKd_{up}(t))-(l_{c}+u_{N})LIKK(t)\\ \;\;\;\;\;\;\;\;\;\;\;\;\;\;\;\;\;\;\;\;\;+d_{N}\;LIKK_{u}(t)+d_{p}\;LIKK_{p}(t)\\ \;\;\;\;\;\;\;\;\;\;\;\;\;\;\;\;\;\;\;\;\;-p_{c}\left(LCBM_{u}(t)+CBMd_{u}(t)\right)LIKK(t)IKK_{tot}\\ \;\;\;\;\;\;\;\;\;\;\;\;\;\;\;\;\;\;\;\;\;-p_{i}\;LIKK(t)\left(LIKK_{u}(t)+IKK_{u}(t)+IKK_{up}(t)+IKKd_{up}(t)\right) \end{array}$$

$$\begin{array}{l} \frac{dLIKK_{u}}{dt}=u_{N}\;LIKK\left(t\right)-\left(d_{N}+l_{C}\right)\;LIKK_{u}\left(t\right)+d_{p}\;LIKK_{up}\left(t\right)\\ \;\;\;\;\;\;\;\;\;\;\;\;\;\;\;\;\;\;\;\;-p_{C}\;\left(LCBM_{u}\left(t\right)+CBMd_{u}\left(t\right)\right)\;LIKK_{u}\left(t\right)\;IKK_{tot}-p_{I}\;LIKK_{u}\left(t\right)\;IKK_{tot} \end{array}$$

$$\frac{dIKK_{u}}{dt}=-p_{C}\left(LCBM_{u}\left(t\right)+CBMd_{u}\left(t\right)\right)\;IKK_{u}\left(t\right)\;IKK_{tot}$$

$$\begin{array}{l} \frac{dIKKd}{dt}=\;l_{C}\;LIKK\left(t\right)+d_{N}\;IKKd_{u}\left(t\right)+d_{p}\;IKKd_{p}\left(t\right)\\ \;\;\;\;\;\;\;\;\;\;\;\;\;\;\;\;\;-p_{C}\;\left(LCBM_{u}\left(t\right)+CBMd_{u}\left(t\right)\right)\;IKKd\left(t\right)\;IKK_{tot}\\ \;\;\;\;\;\;\;\;\;\;\;\;\;\;\;\;\;-p_{I}\;IKKd\left(t\right)\;\left(LIKK_{u}\left(t\right)+IKK_{u}\left(t\right)+IKK_{up}\left(t\right)+IKKd_{up}\left(t\right)\right) \end{array}$$

$$\begin{array}{l} \frac{dIKKd_{u}}{dt}=\;l_{C}\;LIKK_{u}\left(t\right)-d_{N}\;IKKd_{u}\left(t\right)+d_{p}\;IKKd_{up}\left(t\right)\\ \;\;\;\;\;\;\;\;\;\;\;\;\;\;\;\;\;\;-p_{C}\;\left(LCBM_{u}\left(t\right)+CBMd_{u}\left(t\right)\right)\;IKKd_{u}\left(t\right)\;IKK_{tot}-p_{I}\;IKKd_{u}\left(t\right)\;IKK_{tot} \end{array}$$

$$\begin{array}{l} \frac{dIKK_{p}}{dt}=\left(p_{c}(LCBM_{u}(t)+CBMd_{u}(t)\right)(IKK_{tot}-IKK_{u}(t)-LIKK_{u}(t)\\ \;\;\;\;\;\;\;\;\;\;\;\;\;\;\;\;\;\;\;\;-IKK_{p}(t)-IKK_{up}(t)-IKKd(t)-IKKd_{u}(t)-LIKK_{p}(t)-LIKK_{up}(t)\\ \;\;\;\;\;\;\;\;\;\;\;\;\;\;\;\;\;\;\;\;-IKKd_{p}(t)-IKKd_{up}(t))IKK_{tot}\\ \;\;\;\;\;\;\;\;\;\;\;\;\;\;\;\;\;\;\;\;+p_{i}(IKK_{tot}-IKK_{u}(t)-LIKK(t)-LIKK_{u}(t)-IKK_{p}(t)-IKK_{up}(t)\\ \;\;\;\;\;\;\;\;\;\;\;\;\;\;\;\;\;\;\;\;-IKKd(t)-IKKd_{u}(t)-LIKK_{p}(t)-LIKK_{up}(t)-IKKd_{p}(t)\\ \;\;\;\;\;\;\;\;\;\;\;\;\;\;\;\;\;\;\;\;-IKKd_{up}(t))\left(LIKK_{u}(t)+IKK_{u}(t)+IKK_{up}(t)+IKKd_{up}(t)\right) \end{array}$$

$$\begin{array}{l} \frac{dLIKK_{p}}{dt}=p_{C}\;\left(LCBM_{u}\left(t\right)+CBMd_{u}\left(t\right)\right)\;LIKK\left(t\right)\;IKK_{tot}\\ \;\;\;\;\;\;\;\;\;\;\;\;\;\;\;\;\;\;\;\;+p_{I}\;LIKK\left(t\right)\;\left(LIKK_{u}\left(t\right)+IKK_{u}\left(t\right)+IKK_{up}\left(t\right)+IKKd_{up}\left(t\right)\right)\\ \;\;\;\;\;\;\;\;\;\;\;\;\;\;\;\;\;\;\;\;-d_{p}\;LIKK_{p}\left(t\right)+d_{N}\;LIKK_{up}\left(t\right) \end{array}$$

$$\begin{array}{l} \frac{dLIKK_{up}}{dt}=\;p_{C}\;\left(LCBM_{u}\left(t\right)+CBMd_{u}\left(t\right)\right)\;LIKK_{u}\left(t\right)\;IKK_{tot}+p_{I}\;\;LIKK_{u}\left(t\right)\;IKK_{tot}\\ \;\;\;\;\;\;\;\;\;\;\;\;\;\;\;\;\;\;\;\;\;\;\;-\left(d_{p}+d_{N}\right)\;LIKK_{up}\left(t\right) \end{array}$$

$$\begin{array}{l} \frac{dIKK_{up}}{dt}=p_{C}\;\left(LCBM_{u}\left(t\right)+CBMd_{u}\left(t\right)\right)\;IKK_{u}\left(t\right)\;IKK_{tot}+p_{I}\;IKK_{u}\left(t\right)\;IKK_{tot}\\ \;\;\;\;\;\;\;\;\;\;\;\;\;\;\;\;\;\;\;-\left(d_{p}+d_{N}\right)\;IKK_{up}\left(t\right) \end{array}$$

$$\begin{array}{l} \frac{dIKKd_{p}}{dt}=p_{C}\;\left(LCBM_{u}\left(t\right)+CBMd_{u}\left(t\right)\right)\;IKKd\left(t\right)\;IKK_{tot}\\ \;\;\;\;\;\;\;\;\;\;\;\;\;\;\;\;\;\;\;+p_{I}\;IKKd\left(t\right)\;\left(LIKK_{u}\left(t\right)+IKK_{u}\left(t\right)+IKK_{up}\left(t\right)+IKKd_{up}\left(t\right)\right)\\ \;\;\;\;\;\;\;\;\;\;\;\;\;\;\;\;\;\;\;-d_{p}\;IKKd_{p}\left(t\right)+d_{N}\;IKKd_{up}\left(t\right) \end{array}$$

$$\begin{array}{l} \frac{dIKKd_{up}}{dt}=p_{C}\;\left(LCBM_{u}\left(t\right)+CBMd_{u}\left(t\right)\right)\;IKKd_{u}\left(t\right)\;IKK_{tot}+p_{I}\;IKKd_{u}\left(t\right)\;IKK_{tot}\\ \;\;\;\;\;\;\;\;\;\;\;\;\;\;\;\;\;\;\;\;\;-\left(d_{p}+d_{N}\right)\;IKKd_{up}\left(t\right) \end{array}$$

**Table 1:** Parameters for CBM_SM.

Symbol	Value	Unit	Description
k_C_	1.482817	1/µM	CBM-LUBAC association
l_C_	0.770128	1/min	CBM-LUBAC dissociation
u_C_	0.217147	1/min	CBM ubiquitination
d_C_	0.178648	1/min	CBM deubiquitination
k_N_	1.210012	1/µM min	CBM-NEMO association
l_N_	0.813971	1/min	CBM-NEMO dissociation
u_N_	0.04963	1/min	NEMO ubiquitination
d_N_	0.399584	1/min	NEMO deubiquitination
p_C_	0.463834	1/µM^2 min	CBM-mediated IKK phosphorylation
p_I_	0.085297	1/µM min	NEMO-mediated IKK phosphorylation
dp	0.682746	1/min	IKK dephosphorylation
Ltot	0.379065	µM	amount of LUBAC
CBMtot	2.317712	µM	amount of CBM complex
IKKtot	0.178664	µM	amount of IKK

The other model, the CBM detailed model (CBM_DM), focused only on the ubiquitination reaction of the CBM complex to elucidate the mechanisms of timing shift of ubiquitination. CARMA1, BCL10, and MALT1 were distinguished, and the reaction coefficients of the ubiquitination of each protein were compared. The model is characterized by the following equations, and parameters and variables are shown in **Tables 2** and **3**;

$$x_{0}^{\prime }\left(t\right)=-k_{0}\;x_{0}\left(t\right)\;LUBAC\left(t\right)$$

$$\begin{array}{l} LUBAC^{\prime }\left(t\right)=-k_{0}\;x_{0}\left(t\right)\;LUBAC\left(t\right)\\ \;\;\;\;\;\;\;\;\;\;\;\;\;\;\;\;\;\;\;\;\;\;+l_{0}\;\left(x_{1}\left(t\right)+x_{2}\left(t\right)+x_{3}\left(t\right)+x_{4}\left(t\right)+x_{5}\left(t\right)+x_{6}\left(t\right)+x_{7}\left(t\right)+x_{8}\left(t\right)\right) \end{array}$$

$$\begin{array}{l} x_{1}^{\prime }\left(t\right)=k_{0}\;x_{0}\left(t\right)\;LUBAC\left(t\right)-k_{1}\;x_{1}\left(t\right)+l_{1}\;x_{2}\left(t\right)-k_{2}\;x_{1}\left(t\right)+l_{2}\;x_{3}\left(t\right)\;-k_{3}\;x_{1}\left(t\right)\;\\ \;\;\;\;\;\;\;\;\;\;\;\;\;\;\;\;\;\;\;\;+l_{3}\;x_{4}\left(t\right)-l_{0}\;x_{1}\left(t\right) \end{array}$$

$$x_{2}^{\prime }\left(t\right)=k_{1}\;x_{1}\left(t\right)-l_{1}\;x_{2}\left(t\right)-k_{4}\;x_{2}\left(t\right)+l_{4}\;x_{5}\left(t\right)-k_{5}\;x_{2}\left(t\right)+l_{5}\;x_{6}\left(t\right)\;-l_{0}\;x_{2}\left(t\right)$$

$$x_{3}^{\prime }\left(t\right)=k_{2}\;x_{1}\left(t\right)-l_{2}\;x_{3}\left(t\right)-k_{6}\;x_{3}\left(t\right)+l_{6}\;x_{5}\left(t\right)-k_{7}\;x_{3}\left(t\right)+l_{7}\;x_{7}\left(t\right)\;-l_{0}\;x_{3}\left(t\right)$$

$$x_{4}^{\prime }\left(t\right)=k_{3}\;x_{1}\left(t\right)-l_{3}\;x_{4}\left(t\right)-k_{8}\;x_{4}\left(t\right)+l_{8}\;x_{6}\left(t\right)-k_{9}\;x_{4}\left(t\right)+l_{9}\;x_{7}\left(t\right)-l_{0}\;x_{4}\left(t\right)$$

$$x_{5}^{\prime }\left(t\right)=k_{4}\;x_{2}\left(t\right)-l_{4}\;x_{5}\left(t\right)+k_{6}\;x_{3}\left(t\right)-l_{6}\;x_{5}\left(t\right)\;-k_{10}\;x_{5}\left(t\right)+l_{10}\;x_{8}\left(t\right)-l_{0}\;x_{5}\left(t\right)$$

$$x_{6}^{\prime }\left(t\right)=k_{5}\;x_{2}\left(t\right)-l_{5}\;x_{6}\left(t\right)+k_{8}\;x_{4}\left(t\right)-l_{8}\;x_{6}\left(t\right)-k_{11}\;x_{6}\left(t\right)+l_{11}\;x_{8}\left(t\right)-l_{0}\;x_{6}\left(t\right)$$

$$x_{7}^{\prime }\left(t\right)=k_{7}\;x_{3}\left(t\right)-l_{7}\;x_{7}\left(t\right)+k_{9}\;x_{4}\left(t\right)-l_{9}\;x_{7}\left(t\right)-k_{12}\;x_{7}\left(t\right)+l_{12}\;x_{8}\left(t\right)-l_{0}\;x_{7}\left(t\right)$$

$$x_{8}^{\prime }\left(t\right)=k_{10}\;x_{5}\left(t\right)-l_{10}\;x_{8}\left(t\right)+k_{11}\;x_{6}\left(t\right)-l_{11}\;x_{8}\left(t\right)+k_{12}\;x_{7}\left(t\right)-l_{12}\;x_{8}\left(t\right)-l_{0}\;x_{8}\left(t\right)$$

$$y_{1}^{\prime }\left(t\right)=a\;\left(l_{1}\;y_{2}\left(t\right)+l_{2}\;y_{3}\left(t\right)+l_{3}\;y_{4}\left(t\right)\right)+l_{0}\;x_{1}\left(t\right)$$

$$y_{2}^{\prime }\left(t\right)=a\;\left(-l_{1}\;y_{2}\left(t\right)+l_{4}\;y_{5}\left(t\right)+l_{5}\;y_{6}\left(t\right)\right)+l_{0}\;x_{2}\left(t\right)$$

$$y_{3}^{\prime }\left(t\right)=a\;\left(-l_{2}\;y_{3}\left(t\right)+l_{6}\;y_{5}\left(t\right)+l_{7}\;y_{7}\left(t\right)\right)+l_{0}\;x_{3}\left(t\right)$$

$$y_{4}^{\prime }\left(t\right)=a\;\left(-l_{3}\;y_{4}\left(t\right)+l_{8}\;y_{6}\left(t\right)+l_{9}\;y_{7}\left(t\right)\right)+l_{0}\;x_{4}\left(t\right)$$

$$y_{5}^{\prime }\left(t\right)=a\;\left(-l_{4}\;y_{5}\left(t\right)-l_{6}\;y_{5}\left(t\right)+l_{10}\;y_{8}\left(t\right)\right)+l_{0}\;x_{5}\left(t\right)$$

$$y_{6}^{'}(t)=a\;(-l_{5}\;y_{6}(t)-l_{8}\;y_{6}(t)+l_{11}\;y_{8}(t))+l_{0}\;x_{6}(t)$$

$$y_{7}^{'}(t)=a\;(-l_{7}\;y_{7}(t)-l_{9}\;y_{7}(t)+l_{12}\;y_{8}(t))+l_{0}\;x_{7}(t)$$

$$y_{8}^{\prime }\left(t\right)=a\;\left(-l_{10}\;y_{8}\left(t\right)-l_{11}\;y_{8}\left(t\right)-l_{12}\;y_{8}\left(t\right)\right)+l_{0}\;x_{8}\left(t\right)$$

**Table 2:** Parameters for CBM_DM.

Symbol	Value	Unit	Description
*k*_0_	0.578	1/*µ*M min	CBM-LUBAC association
*k*_1_	0.322	1/min	MALT1 of CBM ubiquitination
*l*_1_	0.386	1/min	MALT1 of CBM* deubiquitination
*k*_2_	0.133	1/min	CARMA1 of CBM ubiquitination
*l*_2_	0.121	1/min	CARMA1 of C*BM deubiquitination
*k*_3_	0.106	1/min	BCL10 of CBM ubiquitination
*l*_3_	0.124	1/min	BCL10 of CB*M deubiquitination
*k*_4_	0.362	1/min	CARMA1 of CBM* ubiquitination
*l*_4_	0.105	1/min	CARMA1 of C*BM* deubiquitination
*k*_5_	0.182	1/min	BCL10 of CBM* ubiquitination
*l*_5_	0.0665	1/min	BCL10 of CB*M* deubiquitination
*k*_6_	0.639	1/min	MALT1 of C*BM ubiquitination
*l*_6_	0.146	1/min	MALT1 of C*BM* deubiquitination
*k*_7_	0.184	1/min	BCL10 of C*BM ubiquitination
*l*_7_	0.0213	1/min	BCL10 of C*B*M deubiquitination
*k*_8_	0.717	1/min	MALT1 of CB*M ubiquitination
*l*_8_	0.260	1/min	MALT1 of CB*M* deubiquitination
*k*_9_	0.770	1/min	CARMA1 of CB*M ubiquitination
*l*_9_	0.0904	1/min	CARMA1 of C*B*M deubiquitination
*k_10_*	0.231	1/min	BCL10 of C*BM* ubiquitination
*l_10_*	0.0273	1/min	BCL10 of C*B*M* deubiquitination
*k_11_*	0.634	1/min	CARMA1of CB*M* ubiquitination
*l_11_*	0.0531	1/min	CARMA1 of C*B*M* deubiquitination
*k_12_*	0.334	1/min	MALT1 of C*B*M ubiquitination
*l_12_*	1.07	1/min	MALT1 of C*B*M* deubiquitination

*ubiquitination state.

**Table 3:** Variables in CBM_DM.

Variable	Molecules
*x*_0_	[CBM]
*x*_1_	[CBML]
*x*_2_	[CBM*L]
*x*_3_	[C*BML]
*x*_4_	[CB*ML]
*x*_5_	[C*BM*L]
*x*_6_	[CB*M*L]
*x*_7_	[C*B*ML]
*x*_8_	[C*B*M*L]
*y*_0_	[CBM’]
*y*_1_	[CBM’]
*y*_2_	[CBM*’]
*y*_3_	[C*BM’]
*y*_4_	[CB*M’]
*y*_5_	[C*BM*’]
*y*_6_	[CB*M*’]
*y*_7_	[C*B*M’]
*y*_8_	[C*B*M*’]

*ubiquitination state, apostrophe (’): inactive state.

In this study, parameters were set by using a genetic algorithm (GA) for both CBM_SM and CBM_DM, and the characteristics of each reaction were analyzed. In addition, in CBM_SM, by changing the coefficient for ubiquitinating CBM and the coefficient for ubiquitinating NEMO, the difference between the activation of IKK *via* CBM and the activation of IKK *via* NEMO was clarified.

## Results

### LUBAC Is a Crucial Regulator of TCR-Mediated NF-κB Activation

**Figure 1:**
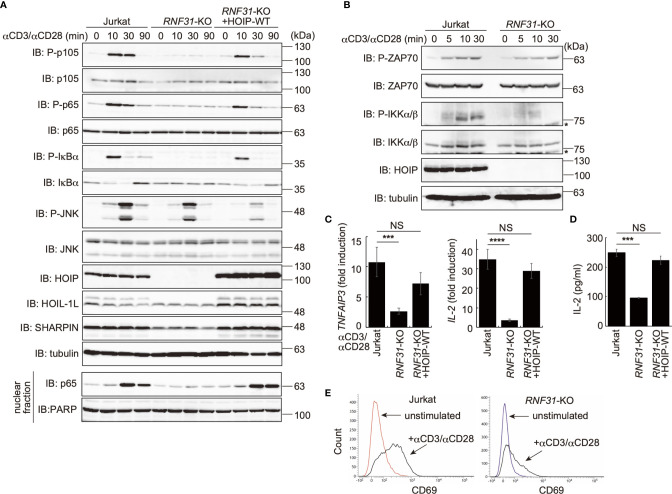
LUBAC is necessary for the TCR-mediated NF-κB activation pathway. **(A)** Parental Jurkat cells, *HOIP*-deficient cells (*RNF31*-KO), or WT-HOIP-restored *RNF31*-KO cells (*RNF31*-KO+HOIP-WT) were stimulated with 1 μg/ml each of anti-CD3 and anti-CD28 antibodies for the indicated periods of time. Cell lysates and nuclear fractions were immunoblotted with the indicated antibodies. **(B)** Impaired IKK activation in *RNF31*-KO cells. WT or *RNF31*-deficient Jurkat cells were stimulated and analyzed as in **(A)**, using the indicated antibodies. *nonspecific signal. **(C)** Reduced expression of TCR-mediated NF-κB target genes in *RNF31*-KO cells. Cells were stimulated with anti-CD3 and anti-CD28 antibodies as in **(A)** for 1 h, and qPCR analyses were performed. **(D)** LUBAC activity is required for efficient IL-2 secretion. Cells were stimulated with 5 μg/ml each of anti-CD3 and anti-CD28 antibodies for 18 h, and secreted IL-2 was quantified by ELISA. **(E)** The expression of T cell activation marker CD69 was suppressed in *RNF31*-KO Jurkat cells. WT or *RNF31*-deficient Jurkat cells were stimulated with 3 μg/ml each of anti-CD3 and anti-CD28 antibodies for 20 h. The expression of CD69 was analyzed by a flow cytometer. **(C, D)** Data are shown as Means ±SD (*n* = 3). ***P<0.001, ****P<0.0001, NS, not significant.

To investigate the involvement of LUBAC in TCR-mediated NF-κB activation, we previously constructed HOIP-deficient human leukemic T cell lymphoblast Jurkat cells (*RNF31*-KO) (39). Moreover, wild type (WT)-HOIP was restored in *RNF31*-KO cells to construct *RNF31*-KO+HOIP-WT cells, to exclude the possibility of off-target depletion. These cells were stimulated with anti-CD3 and anti-CD28 antibodies, and TCR-mediated NF-κB activation was examined (**Figure 1A**). Although efficient phosphorylation of the canonical NF-κB factors, such as p105, p65 and IκBα, degradation and regeneration of IκBα, and intranuclear translocation of p65 were detected in parental Jurkat and *RNF31*-KO+HOIP-WT cells upon TCR-stimulation, NF-κB activation was markedly suppressed in *RNF31*-KO cells. These results suggested that the LUBAC activity is indispensable for the TCR-mediated NF-κB activation. Importantly, the tyrosine kinase ZAP70, which associates with the TCRζ chain and is activated upon stimulation (27), was similarly phosphorylated in *RNF31*-KO and the parental Jurkat cells upon TCR stimulation (**Figure 1B**). In contrast, the phosphorylation of IKKα/β, which represents IKK activation, was strongly impaired in *RNF31*-KO cells. Thus, the LUBAC activity is involved downstream from ZAP70, and upstream from IKK activation. In the absence of LUBAC activity, the expression of NF-κB targets, such as *TNFAIP3* (which encodes A20), *interleukin-2*(*IL-2*), and *NFKBIA* (IκBα), and secreted IL-2 were suppressed after TCR stimulation by anti-CD3 and anti-CD28 antibodies or a combined treatment with PMA and ionomycin, which mimics TCR stimulation (**Figures 1C, D**, **Supplementary Figure 1**). As a result, the expression of CD69, a T cell activation marker, was suppressed in TCR-stimulated *RNF31*-KO cells (**Figure 1E**). Collectively, these results indicated that LUBAC plays a pivotal role in the TCR-mediated NF-κB activation and T cell activation.

### LUBAC Binds CBM Complex Through HOIP

**Figure 2:**
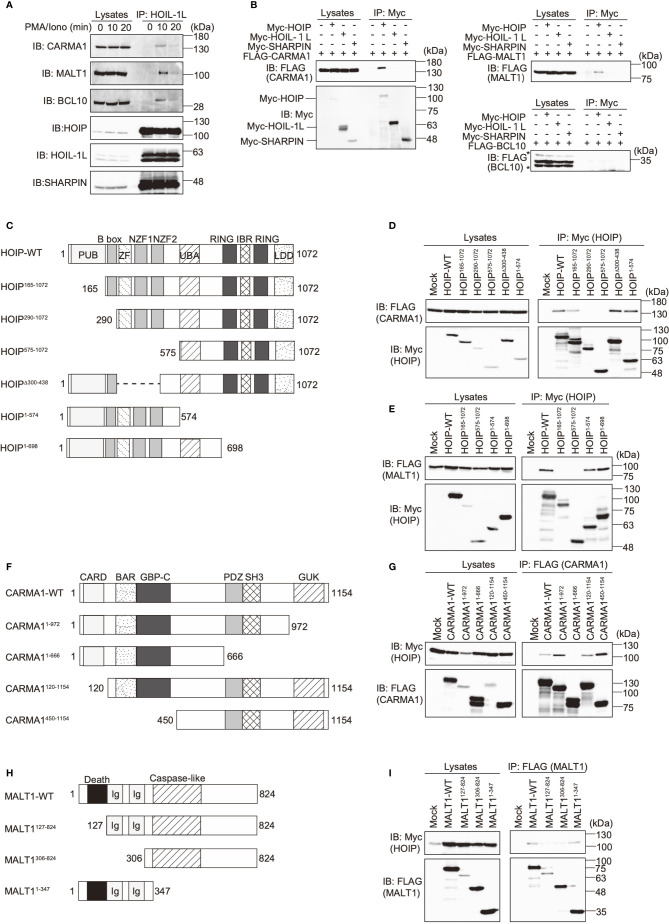
The N-terminal domains in HOIP associate with CARMA1 and MALT1. **(A)** Endogenous association of LUBAC and the CBM complex upon TCR-stimulation. Jurkat cells were stimulated with 20 ng/ml PMA and 150 ng/ml ionomycin for the indicated time periods. The cells lysates and anti-HOIL-1L immunoprecipitates were subjected to immunoblotting with the indicated antibodies. **(B)** HOIP binds CARMA1 and MALT1. Myc-tagged LUBAC subunits and FLAG-tagged CBM components were co-expressed in HEK293T cells, as indicated. The cell lysates and anti-Myc immunoprecipitates were immunoblotted with the depicted antibodies. **(C)** Domain structure and mutants of HOIP. PUB: peptide:N-glycanase/UBA or UBX-containing proteins; ZF: zinc finger; NZF: Npl4-type zinc finger; UBA: ubiquitin-associated; RING: really interesting new gene; IBR: in-between RING; and LDD: linear ubiquitin determining. **(D)** The B box domain of HOIP is crucial for CARMA1-binding. WT and various mutants of Myc-tagged HOIP were co-expressed with FLAG-CARMA1 in HEK293T cells, and immunoprecipitations followed by immunoblotting analyses were performed as indicated. **(E)** The PUB domain of HOIP is responsible for MALT1-binding. A similar analysis to that in **Figure 2D** was performed, using FLAG-MALT1. **(F)** Domain structure and mutants of CARMA1. CARD, caspase-recruitment domain; BAR, Bin/Amphiphysin/Rvs; GBP-C, guanylate-binding protein C‐terminal; PDZ, post synaptic density protein (PSD95)-Drosophila disc large tumor suppressor (Dlg1)-Zonula occludens-1 protein (ZO-1); SH3, Src homology 3; and GUK, guanylate kinase. **(G)** The PDZ-SH3 region of CARMA1 binds HOIP. A similar analysis to that in **Figure 2D** was performed, using WT and various mutants of FLAG-CARMA1 and Myc-HOIP. **(H)** Domain structure and mutants of MALT1. Ig, immunoglobulin-like. **(I)** The death domain in MALT1 is the HOIP binding site. A similar analysis to that in **Figure 2D** was performed, using WT and various mutants of FLAG-MALT1 and Myc-HOIP.

To investigate the involvement of LUBAC in the TCR pathway, we analyzed the interaction of LUBAC with the CBM complex. Upon stimulation with PMA/ionomycin, the endogenous LUBAC transiently binds with the CBM complex in Jurkat cells (**Figure 2A**). Similarly, TCR stimulation by anti-CD3 and anti-CD28 antibodies also enhanced the association of endogenous LUBAC and the CBM complex in Jurkat cells (**Supplementary Figure 2A**). Co-immunoprecipitation followed by immunoblotting experiments indicated that HOIP, but not HOIL-1L or SHARPIN, bound to CARMA1 and MALT1, whereas the direct binding of LUBAC subunits with BCL10 was negligible (**Figure 2B**). To identify the binding sites in HOIP that interact with CARMA1 and MALT1, various HOIP mutants were constructed (**Figure 2C**). The results revealed that the B box (aa 165–289) and PUB (aa 1–164) domains in HOIP are the binding sites for CARMA1 and MALT1, respectively (**Figures 2D, E**). Moreover, the PDZ and SH3 regions (aa 667–972) of CARMA1 (**Figures 2F, G**) and the N-terminal death domain (aa 1–126) of MALT1 were identified as HOIP-binding sites (**Figures 2H, I**). We further performed transient co-expression followed by an immunoprecipitation analysis to investigate the LUBAC-CBM complex, using WT or HOIP-binding region-deleted mutants of CARMA1 and/or MALT1 (**Supplementary Figure 2B**). The results revealed that the CBM containing the HOIP-binding region-deleted CARMA1^1–666^ or MALT1^127–824^ failed to associate with the LUBAC complex, suggesting that the HOIP-binding sites in CARMA1 and MALT1 are indispensable for the association of the CBM complex with LUBAC.

**Figure 3:**
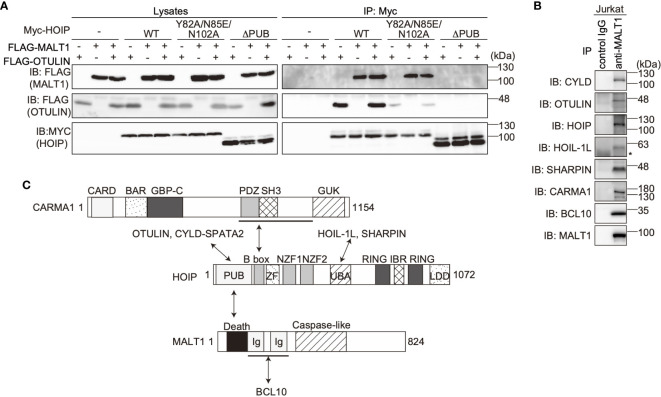
OTULIN and MALT1 simultaneously bind to the PUB domain of HOIP. **(A)** WT and various mutants of Myc-HOIP were co-expressed with FLAG-MALT1 and/or FLAG-OTULIN in HEK293T cells, as indicated. Cell lysates and anti-Myc immunoprecipitates were immunoblotted with the indicated antibodies. **(B)** The endogenous association of CYLD, OTULIN, LUBAC, and the CBM complex. Jurkat cell lysates were immunoprecipitated with normal mouse IgG or anti-MALT1 antibody, and subjected to immunoblotting by the depicted antibodies. *; nonspecific signal. **(C)** Schematic illustration of the LUBAC/DUBs-CBM complex interaction.

The PUB domain of HOIP reportedly binds p97 and DUBs, such as OTULIN and CYLD-SPTA2 (16, 17), and here we determined that it also binds to the death domain of MALT1. To examine whether MALT1 binds the PUB domain of HOIP in a mutually exclusive manner with OTULIN, immunoprecipitation followed by immunoblotting analyses were performed using PUB mutants (**Figure 3A**). Both MALT1 and OTULIN simultaneously bound the WT-HOIP. In contrast, the Y82A/N85E/N102A mutant of HOIP, in which the critical OTULIN-binding residues are replaced (16, 17), drastically reduced the OTULIN binding, although MALT1 binding was not affected. Moreover, neither MALT1 nor OTULIN could bind the PUB domain-deleted mutant of HOIP. These results indicated that MALT1 and OTULIN bind the PUB domain of HOIP in independent manners. Furthermore, we identified the endogenous association of CYLD and OTULIN with LUBAC and the CBM complex by the immunoprecipitation with anti-MALT1 antibody (**Figure 3B**). Collectively, these results suggested that LUBAC physiologically associates with DUBs at the HOIP PUB domain, and further binds the CBM complex through HOIP upon TCR stimulation to form signaling complex (**Figure 3C**).

### Linear Ubiquitination of CBM Complex Predominantly Induces TCR-Mediated NF-κB Activation

**Figure 4:**
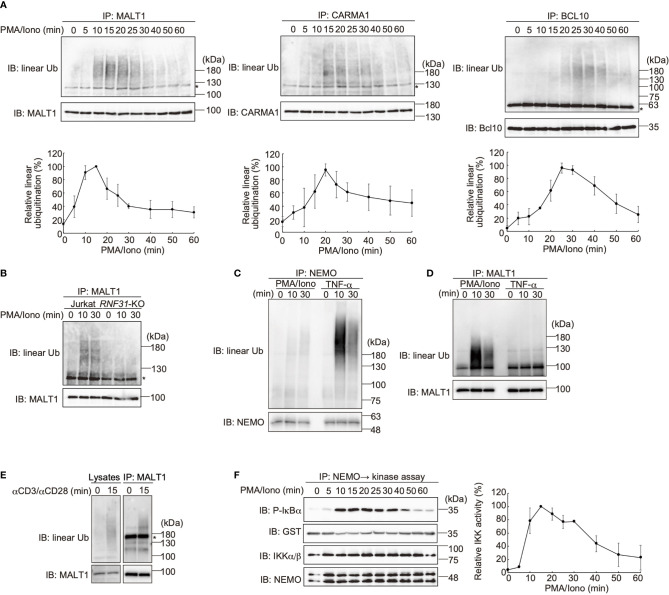
The CBM complex is linearly ubiquitinated upon TCR stimulation. **(A)** Parental Jurkat cells were stimulated with 20 ng/ml PMA and 150 ng/ml ionomycin for the indicated time periods. The heat-denatured cell lysates were immunoprecipitated and immunoblotted with the indicated antibodies. Taking the maximum intensities of linear ubiquitination as 100%, the relative intensities of the linear ubiquitinated CBM complex are indicated. Means ±SD (*n* = 3). **(B)** HOIP is required for linear ubiquitination of MALT1. Parental and *RNF31*-KO Jurkat cells were stimulated with PMA/ionomycin as in **(A)**, immunoprecipitated with anti-MALT1, and immunoblotted with the indicated antibodies. **(C)** Suppressed linear ubiquitination of NEMO in the TCR-mediated NF-κB activation pathway. Jurkat cells were stimulated with 20 ng/ml PMA and 150 ng/ml ionomycin or 1 µg/ml TNF-α for the indicated time periods, and cell lysates were immunoprecipitated with an anti-NEMO antibody and then immunoblotted with the depicted antibodies. **(D)** MALT1 is exclusively linearly ubiquitinated upon TCR stimulation. A similar analysis to that in **Figure 4C** was performed after anti-MALT1 immunoprecipitation. **(E)** Linear ubiquitination of MALT1 after stimulation with CD3 and CD28. Jurkat cells were stimulated with 5 µg/ml each of anti-CD3 and anti-CD28 antibodies as indicated. The cell lysates and anti-MALT1 immunoprecipitates were immunoblotted by the indicated antibodies. **(F)** Transient activation of canonical IKK. Jurkat cells were stimulated with 20 ng/ml PMA and 150 ng/ml ionomycin for the indicated time periods. After immunoprecipitation with an anti-NEMO antibody, an *in vitro* canonical IKK assay was performed using GST-IκBα^1–54^ as the substrate. Samples were immunoblotted with the indicated antibodies, and taking the maximum intensities of P-IκBα as 100%, the relative intensities are indicated. Means ±SD (*n* = 3). **(A, B, E)** *; nonspecific signal.

LUBAC reportedly linearly ubiquitinates BCL10 in the CBM complex (32), whereas the effects of LUBAC on CARMA1 and MALT1 have not been clarified. Therefore, we performed SDS-hot lysis followed by immunoprecipitation and immunoblotting analyses. Unexpectedly, all of the CBM subunits were transiently linearly ubiquitinated after PMA/ionomycin stimulation of Jurkat cells with different time courses. Thus, MALT1 was initially linearly ubiquitinated after 15 min as the maximum, and subsequently, CARMA1 (20 min) and BCL10 (25 min) were linearly ubiquitinated (**Figure 4A**). Indeed, the TCR-induced linear ubiquitination of MALT1 was abolished in *RNF31*-KO cells (**Figure 4B**), indicating that the LUBAC activity is necessary for the linear ubiquitination.

We previously showed that NEMO is a physiological substrate of LUBAC upon TNF-α and IL-1β stimulation (40), and it functions as a scaffold to recruit other IKK molecules *via* its UBAN domain, which specifically binds to linear ubiquitin chains (10). The recruited and concentrated IKK molecules are then activated by *trans*-phosphorylation (11). Indeed, NEMO was efficiently linearly ubiquitinated upon TNF-α stimulation in Jurkat cells, whereas the linear ubiquitination of NEMO was suppressed after PMA/ionomycin-treatment (**Figure 4C**). In contrast, MALT1 was linearly ubiquitinated by a PMA/ionomycin-treatment, but not by TNF-α stimulation (**Figure 4D**). Not only PMA/ionomycin-treatment, but also the linear ubiquitination of endogenous MALT1 was detected after stimulation with anti-CD3 and anti-CD28 antibodies (**Figure 4E**). These results suggested that the CBM complex is the major substrate of LUBAC during TCR-mediated NF-κB activation. To examine the canonical IKK activity, Jurkat cells were treated with PMA/ionomycin, and afterwards the endogenous NEMO was immunoprecipitated and subjected to an *in vitro* canonical IKK assay, using GST-IκBα as a substrate (**Figure 4F**). The IκBα phosphorylation reached the maximum after 15 min of stimulation, and declined thereafter. Collectively, these results suggested that the linear ubiquitination of the CBM complex by LUBAC correlates with the canonical IKK activation in the TCR-mediated NF-κB activation pathway.

### OTULIN Is the Predominant Regulator of TCR-Mediated NF-κB Activation

**Figure 5:**
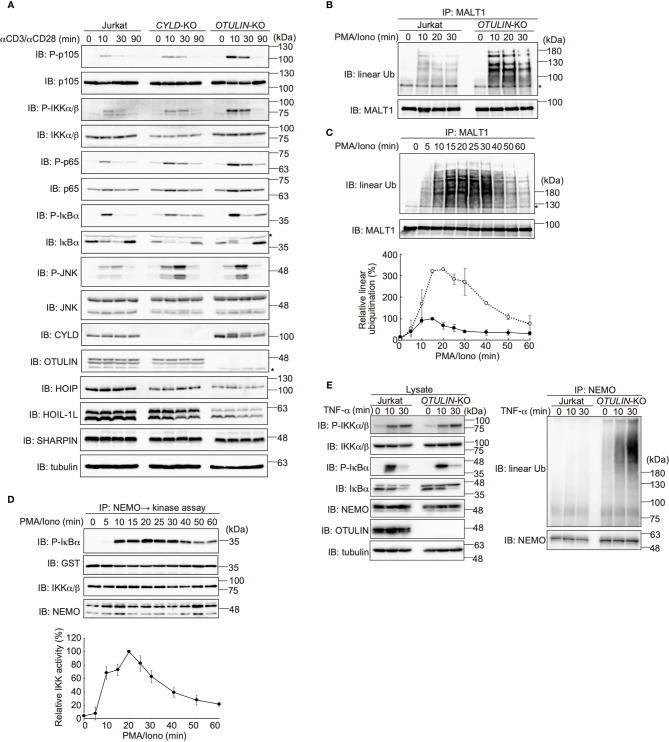
OTULIN predominantly downregulates TCR-mediated NF-κB activation in Jurkat cells. **(A)** Parental, *CYLD*-deficient (*CYLD*-KO), and *OTULIN*-deficient (*OTULIN*-KO) Jurkat cells were stimulated with 4 μg/ml each of anti-CD3 and anti-CD28 antibodies for the indicated time periods, and cell lysates were immunoblotted with the depicted antibodies. **(B)** Augmented linear ubiquitination of MALT1 in *OTULIN*-deficient cells. Parental and *OTULIN*-KO Jurkat cells were stimulated with 20 ng/ml PMA and 150 ng/ml ionomycin for the indicated time periods, and the linear ubiquitination of MALT1 was compared as in **Figure 4A**. **(C)** Semi-quantification of MALT1 linear ubiquitination in *OTULIN*-KO cells. *OTULIN*-KO cells were stimulated with PMA and ionomycin, and analyzed as in **Figure 4A**. Taking the maximum intensities of the linear ubiquitination of MALT1 in parental Jurkat cells as 100% (*closed circles*), the relative intensities of linearly ubiquitinated MALT1 in *OTULIN*-KO cells are indicated (*open circles*). Means ±SD (*n* = 3). **(D)** Transient activation of canonical IKK in *OTULIN*-KO cells. *OTULIN*-KO cells were stimulated with PMA and ionomycin, and analyzed as in **Figure 4E**. **(E)** Enhanced linear ubiquitination of NEMO in *OTULIN*-KO cells upon TNFα treatment. Jurkat and *OTULIN*-KO cells were stimulated with 1 µg/ml TNF-α for the indicated time periods, and analyzed as in **Figure 4C**. **(A–C)** *; nonspecific signal.

DUBs, such as OTULIN and the CYLD-SPATA2 complex, bind to the PUB domain of HOIP and downregulate NF-κB activity by hydrolyzing the ubiquitin chains (16–18). To examine the effect of these DUBs on the TCR-mediated NF-κB activation, we constructed *CYLD*- and *OTULIN*-deficient Jurkat cells (**Supplementary Figure 3**). Upon stimulation with anti-CD3 and anti-CD28 antibodies, the phosphorylation of NF-κB factors, such as p105, IKKα/β, p65, and IκBα, was enhanced in *OTULIN*-KO cells as compared with those in parental cells (**Figure 5A**). In contrast, similar activation of NF-κB signaling factors was detected in *CYLD*-KO cells. Interestingly, the phosphorylation of JNK, a MAP kinase, was enhanced in both *CYLD*- and *OTULIN*-KO cells. The restoration of OTULIN-WT in *OTULIN*-KO cells canceled the enhanced phosphorylation of NF-κB signaling factors in *OTULIN*-KO cells stimulated with anti-CD3 and anti-CD28 antibodies (**Supplementary Figure 4**). These results clearly indicated that OTULIN down-regulates the TCR-mediated NF-κB activation pathway.

In *OTULIN*-KO cells, linear ubiquitination of MALT1 was augmented ~3-fold over that of the parental Jurkat cells (**Figures 5B, C**). In *OTULIN*-deficient cells, canonical IKK was transiently activated with a time course similar to that of the parental cells (**Figure 5D**). Moreover, in *OTULIN*-KO Jurkat cells, the enhanced linear ubiquitination of NEMO was detected after TNF-α stimulation (**Figure 5E**). These results suggested that OTULIN, but not CYLD, plays a major role in the downregulation of the LUBAC-mediated canonical NF-κB activation pathways in Jurkat cells.

### Mathematical Model for Linear Ubiquitination-Mediated IKK Activation in TCR Pathway

**Figure 6:**
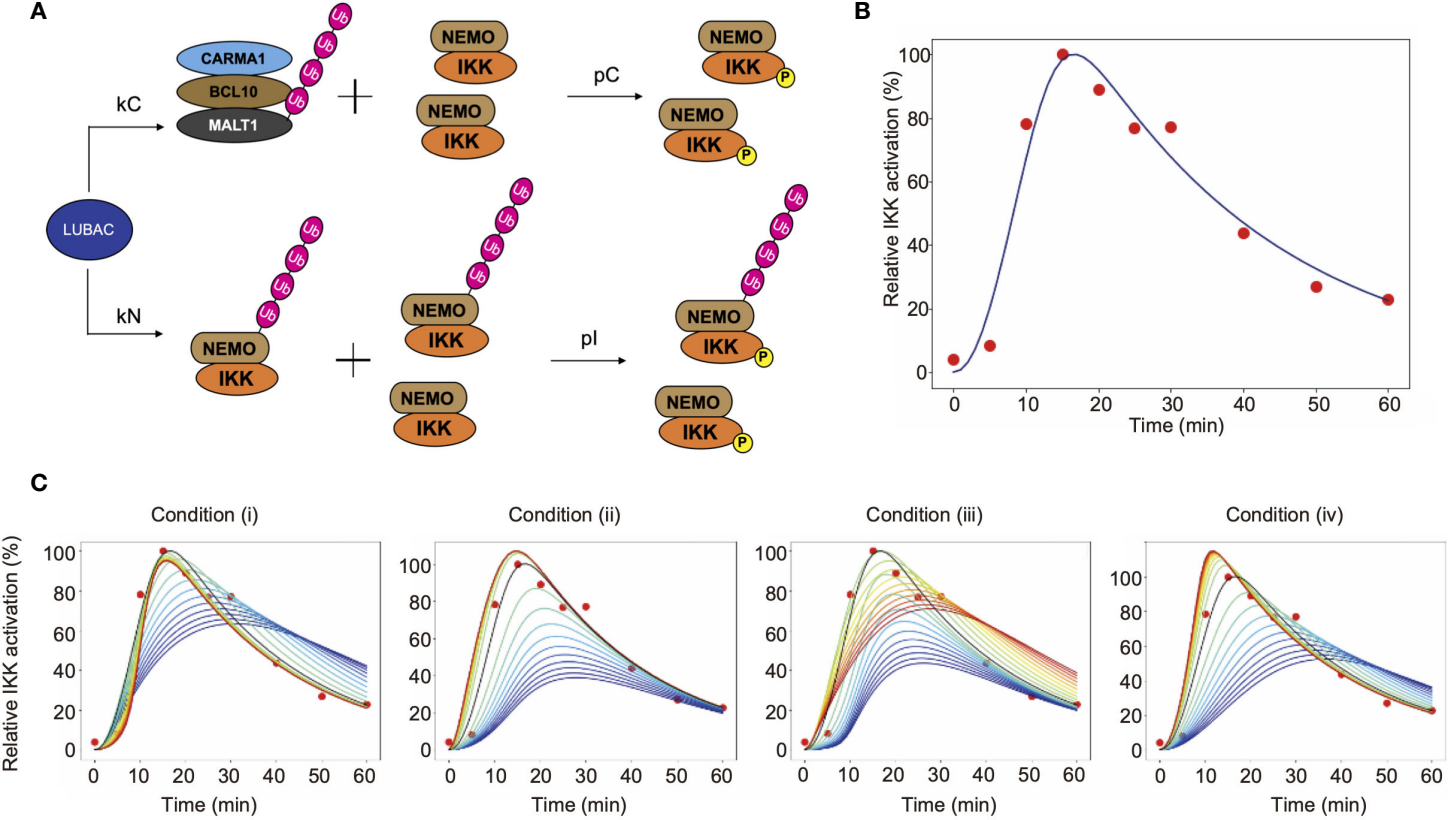
Mathematical simulation of the effects of linear ubiquitination of the CBM complex and NEMO on the TCR-mediated IKK activation. **(A)** LUBAC ubiquitinates NEMO and the CBM complex. In this model, ubiquitination is assumed to be a first-order reaction. **(B)** Fitting result by GA. Solid lines represent simulation results, and dots represent experimental data. **(C)** The simulation results of IKK activation, changing *k_C_* and *k_N_*. From the blue line to the red line, *k_C_* increases under condition (i), and *k_N_* increases under condition (ii). Condition (iii) is a simulation result when *θ* = π/4. From the blue line to the red line, *k_C_* increases and *k_N_* decreases. Condition (iv) is a simulation result when *θ* = 3π/4. From the blue line to the red line, *k_C_* decreases and *k_N_* increases.

To investigate the characteristics of LUBAC in the TCR-mediated NF-κB activation pathway, we mathematically considered the reaction of LUBAC-mediated IKK activation through the linear ubiquitination of NEMO and CBM (**Figure 6A**). Since IKK is *trans*-activated by using the linear ubiquitin chain as a scaffold, the NEMO-mediated activation of IKK occurs between ubiquitinated IKKs or between ubiquitinated IKKs and non-ubiquitinated IKKs. On the other hand, the CBM-mediated activation of IKK occurs by contact between ubiquitinated CBM and IKKs that are not distinguished by their ubiquitination state. In addition, the mass conservation law for the LUBAC and CBM complex holds, because protein production and degradation are not considered.

The parameters of CBM_SM were set by a genetic algorithm, using experimental data of the ubiquitinated CBM complex and phosphorylated IKK obtained in **Figures 4** and **5** (**Figure 6B**, **Table 1**). The estimation was performed 1,000 times with the setting to generate 1,000 generations. We set the estimation results such that the error from the experimental data is small and the concentrations of LUBAC, CBM, and IKK were close to the concentrations of general signal transduction factors (0.1 µM). However, all of the parameter settings were values larger than 0.1 µM, since proteins with different molecular weights accumulate and are locally concentrated on the linear ubiquitin chains, and T cells are smaller than general somatic cells. Importantly, the parameters obtained by the GA showed that the CBM complex is more likely to bind to LUBAC than NEMO (k_C_>k_N_). Moreover, the ubiquitination rate of the CBM complex was faster than that of NEMO (u_C_>u_N_). These results suggested that IKK activation induced by the linear ubiquitination of the CBM complex plays a major role to activate IKK in T cells, by the linear ubiquitination of NEMO.

To quantitatively analyze the effect of the CBM complex on IKK activation, we performed a simulation by changing the binding rate of LUBAC and CBM (k_C_) or NEMO (k_N_). The simulation was performed under the following four conditions (where k_CG_ and k_NG_ mean the parameter set in GA) (**Figure 6C**).

(i) Fix k_N_ and change k_C_

$$k_{N}=k_{NG}$$

$$k_{C}=r\;k_{CG}\;(r=0.1,\;0.2,\cdots ,0.9,\;1,\;2,\cdots ,\;9,\;10)$$

(ii) Fix k_C_ and change k_N_

$$k_{C}=k_{CG}$$

$$k_{N}=r\;k_{NG}\;(r=0.1,\;0.2,\cdots ,0.9,\;1,\;2,\cdots ,\;9,\;10)$$

(iii) Change k_C_ and k_N_ simultaneously in inverse proportion

$$k_{C}=k_{CG}/\left(1+r\cos\theta \right)\left(r=\;1,\;2,\cdots ,\;9,\;10\right)$$

$$k_{N}=\left(1+r\sin\theta \right)\;k_{NG}\;\left(r=\;1,\;2,\cdots ,\;9,\;10\right)$$

and

$$k_{C}=\left(1+r\cos\theta \right)k_{CG}\left(r=\;1,\;2,\cdots ,\;9,\;10\right)$$

$$k_{N}=\;k_{NG}/\left(1+r\sin\theta \right)\left(r=\;1,\;2,\cdots ,\;9,\;10\right)$$

$$\left(\frac{\pi }{2}\leq \theta <\pi \right)$$

(iv) Change k_C_ and k_N_ proportionally at the same time

$$k_{C}=\left(1+r\cos\theta \right)k_{CG}\;\left(r=1,\;2,\cdots ,\;9,\;10\right)$$

$$k_{N}=\left(1+r\sin\theta \right)\;k_{NG}\;\left(r=\;1,\;2,\cdots ,\;9,\;10\right)$$

and

$$k_{C}=k_{CG}/\left(1+r\cos\theta \right)\left(r=1,\;2,\cdots ,\;9,\;10\right)$$

$$k_{N}=\;k_{NG}/\left(1+r\sin\theta \right)\left(r=\;1,\;2,\cdots ,\;9,\;10\right)$$

$$0\leq \theta <\frac{\pi }{2}$$

From the simulation results (i) and (iii), when k_C_ is large, the IKK activation peak rises quickly. Immediately after stimulation, NEMO-mediated activation occurs earlier, but when the effect of CBM-mediated activation is greater, the peak maximum is reached sooner. This is because all IKKs can be activated *via* CBM, without depending on the ubiquitination state of NEMO. In addition, DUBs are stabilized by the ubiquitin chain of CBM, and thus CBM is rapidly deubiquitinated. Since the activation of IKK is also reduced, IKK can rapidly switch its active state *via* CBM. Furthermore, from the results of simulation (ii), the activation level decreases when k_N_ is small. In other words, these results suggest that the NEMO-mediated activation of IKK is responsible for the strength of the response to the stimulus. The results from simulation (iv) revealed that when the ubiquitination of both CBM and NEMO is suppressed, the activation of IKK also decreases, and the peak timing is delayed. This reflects the need for rapid responses to external stimuli in both pathways.

### Mathematical Simulation for Linear Ubiquitination of CBM Complex Components

**Figure 7:**
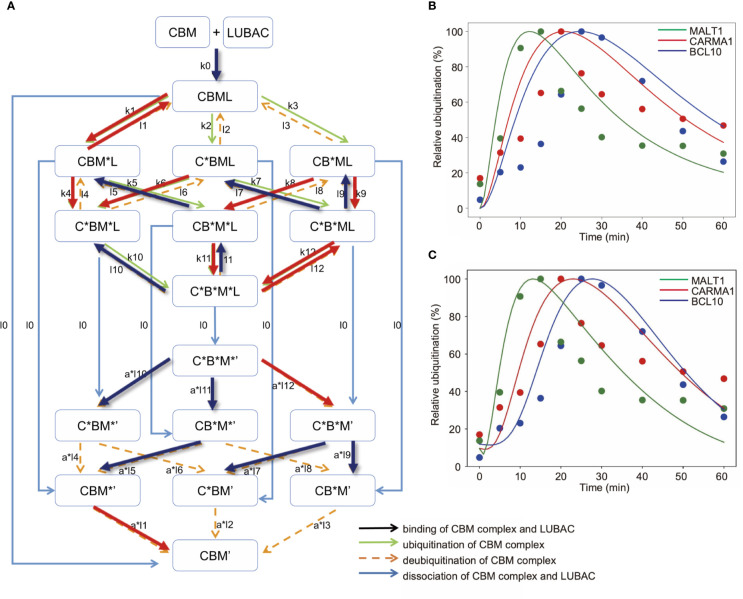
Mathematical model for different linear ubiquitinations of the CBM complex. **(A)** Detailed reaction pathway of the ubiquitination of the CBM complex. The red lines show parameters that are larger than the average value. The blue lines show parameters that are smaller than the average value. **(B)** Fitting results by GA in the CBM_DM. **(C)** Fitting results by GA. The ubiquitination reaction is assumed to be a Hill function.

Finally, we constructed an expanded model to analyze the timing deviations in the linear ubiquitinations of CARMA1, BCL10, and MALT1. By focusing on the linear ubiquitination of the CBM complex, we constructed a model that distinguishes CARMA1, BCL10, and MALT1. The amounts of ubiquitinated proteins were then simulated. If the ubiquitination and deubiquitination rates of CARMA1, BCL10, and MALT1 were the same values, then the CBM_DM is represented by the same model as the CBM_SM, by equating CARMA1, BCL10, and MALT1. In this model, we assumed that ubiquitination is a first-order reaction for simplicity (**Figure 7A**).

As a result of fitting with the GA, the timing shifts of the peaks of MALT1, CARMA1, and BCL10 could be reproduced (**Figure 7B**). By focusing on the parameters, we found that MALT1, CARMA1, and BCL10 have different ubiquitination thresholds (**Tables 2**, **3**). One of the reasons could be that the ubiquitin chain is extended, thus further stabilizing the ubiquitination. In addition, the ubiquitinating and deubiquitinating enzymes may evaluate the states of MALT1, CARMA1, and BCL10. On the other hand, in this simulation, the increase and decrease of ubiquitination levels did not match well. Therefore, more detailed modeling of ubiquitination is needed to solve this problem. Since ubiquitin is consecutively linked and its activity changes depending on its length, ubiquitination cannot be expressed well by the assumption of the first-order reaction. The model could be improved by considering the production levels of linear (de)-ubiquitination of MALT1, CARMA1, and BCL10 as a switch-like reaction; for example, by applying the Hill equation (**Figure 7C**). These results indicated that length of the ubiquitin-linkage allosterically regulates the generation and degradation of linear ubiquitin chains.

## Discussion

T lymphocytes play crucial roles in the host defence against pathogens and tumors, and TCR recognizes the MHC-bound antigen peptide fragments derived from them (26). In this study, we initially identified that LUBAC is indispensable for the TCR-mediated NF-κB pathway and IL-2–mediated T cell activation (**Figure 1**). LUBAC physiologically associates with the CBM complex, and the PUB and B-box domains of HOIP bind the death domain in MALT1 and the PDZ-SH3 region in CARMA1, respectively (**Figure 2**). The binding of MALT1 to the HOIP PUB domain did not disturb the association with OTULIN (**Figure 3**), indicating that MALT1 simultaneously binds LUBAC with DUBs, such as OTULIN and CYLD-SPATA2, through the HOIP PUB domain. The crystal structure of the PUB-interacting motif (PIM) of OTULIN with the HOIP PUB domain revealed that several residues, such as Tyr82, Asn85, Asn101, and Asn102, form hydrogen-bonds with the OTULIN PIM (16). Importantly, a patient with the Leu72→Pro missense mutation in the HOIP PUB domain reportedly exhibited multiorgan autoinflammation, immunodeficiency, amylopectinosis, and lymphangiectasia with impaired distributions and functions of T lymphocytes (41), suggesting that the effects of the mutation may be due to the dysfunctional binding of LUBAC to the CBM complex. Importantly, the B-box domain in CYLD is involved in the intermolecular interaction (42), and the B-box-containing region in HOIP reportedly affected the dimerization of HOIP (16). Therefore, the deletion of this region may affect the architecture of LUBAC, resulting in defective CBM complex binding. Further detailed structural analyses will be necessary for clarification.

At present, various E3s, such as c-IAP1/2, Cbl-B, STUB1, NEDD4, ITCH, RNF181, TRAF6, and LUBAC, reportedly ubiquitinate the CBM components in the TCR- and BCR-signaling pathways (43). Although LUBAC is dispensable for the BCR-mediated NF-κB activation pathway, crosstalk between LUBAC and the CBM complex in B cells has been reported. Upon BCR stimulation, BCL10 is linearly ubiquitinated by LUBAC in a TRAF6-induced K63-ubiquitination-dependent manner (31). Importantly, the Q622L polymorphism in HOIP, which enhances the LUBAC activity, is associated with activated B cell-like diffuse large B cell lymphoma (ABC-DLBCL) (44), and c-IAP-1/2–mediated K63-ubiquitination is involved in the LUBAC recruitment and the linear ubiquitination of BCL10 in ABC-DLBCL cells (45). Although some controversy exists regarding the necessity of the LUBAC catalytic activity for ABC-DLBCL pathogenesis (30), LUBAC is crucial for B cell lymphomagenesis through protection against DNA damage-induced cell death (46). Therefore, the suppression of LUBAC activity is a suitable therapeutic target for ABC-DLBCL (44, 46, 47).

In this study, we identified that in addition to BCL10, MALT1 and CARMA1 are also linearly ubiquitinated by LUBAC (**Figure 4**), suggesting that the CBM complex components are physiological substrates for LUBAC. Among them, the linear ubiquitination of MALT1 seems to precede the canonical IKK activation. In the case of MALT1, TRAF6-mediated K63-ubiquitination at the C-terminal portion of Lys residues is reportedly involved in NEMO recruitment, NF-κB activation, and IL-2 production (48). Subsequently, A20, a DUB, removes the K63-ubiquitin chain from MALT1 and thus downregulates the NF-κB activation pathway (49). Moreover, the monoubiquitination of K644 in MALT1 by an unknown E3 is required for the constitutive protease activity of MALT1 (50). Interestingly, the paracasapase activity of MALT1 cleaves the Arg^165^-Gly^166^ bond of HOIL-1L, and thus abrogates the linear ubiquitination activity of LUBAC in B cells (51). We could not detect the MALT1-induced HOIL-1L cleavage upon TCR stimulation in Jurkat cells during the experimental incubation period (~90 min); however, prolonged TCR stimulation may affect the proteostasis of LUBAC.

We determined that OTULIN, but not CYLD, plays a pivotal role in the suppression of TCR-mediated NF-κB activation in Jurkat cells, although JNK was activated in either *OTULIN*- or *CYLD*-deficiency (**Figure 5**). A homozygous mutation in the *OTULIN* gene causes autoinflammation, named OTULIN-related autoinflammatory syndrome (ORAS), which induces the degradation of LUBAC subunits in T and B cells (52). Moreover, TCR-induced JNK activation is required for the MALT1-mediated proteolytic inactivation at Arg324 of CYLD (53). Thus, these DUBs are involved in the crosstalk regulation of the TCR-mediated NF-κB activation pathway.

The mathematical analysis of the NF-κB signaling pathway has provided a novel paradigm for spatiotemporal activation mechanism, target gene expression, feed-back inhibition, and cytosol-nucleus oscillation of the transcription factor, and various mathematical models have been proposed (35–37). In contrast to TNF-α-mediated NF-κB activation pathway, the CBM complex, but not NEMO, was preferentially ubiquitinated by LUBAC upon TCR stimulation (**Figure 4**), and the mathematical analysis indicated that linear ubiquitination of the CBM complex stably promotes IKK activation (**Figure 6**). On the other hand, NEMO-mediated activation of IKK was required to increase the activation level of IKK. In addition, we identified the differences in the timing of ubiquitination between CARMA1, BCL10, and MALT1 in the CBM complex (**Figure 4**), although its physiological function was not clear. The mathematical modeling suggested that by shifting the timing of the MALT1, CARMA1, and BCL10 ubiquitinations, the scaffolding of ubiquitin chains persists, and IKK can be stably activated due to the allosteric regulation (**Figure 7**). Moreover, DUBs, such as OTULIN, can quickly downregulate IKK and then restore it to the original state. Thus, these mathematical simulations were effective in characterizing the experimentally obtained features in TCR-mediated NF-κB pathway. However, in order to quantitatively analyze the timing shifts of the CARMA1, BCL10, and MALT1 ubiquitinations, a model that reflects the detailed mechanism of ubiquitination will be required.

## Data Availability Statement

The original contributions presented in the study are included in the article/**Supplementary Material**. Further inquiries can be directed to the corresponding authors.

## Ethics Statement

The protocols were approved by the Safety Committee for Recombinant DNA Experiments and the Safety Committee for Bio-Safety Level 2 (BSL-2) Experiments of Osaka City University.

## Author Contributions

DO performed cell biological experiments. NH performed the mathematical simulation. TS and FT coordinated the study. All authors wrote and commented on the manuscript, and discussed the results. All authors contributed to the article and approved the submitted version.

## Funding

This work was partly supported by MEXT/JSPS KAKENHI grants (Nos. JP16H06276 (AdAMS), JP16H06575, JP18H02619, and JP19K22541 to FT, 16H06576 to TS, and JP18K06967, JP19H05296, and 20H5337 to DO), Takeda Science Foundation (FT), a Grant for Research Program on Hepatitis from the Japan Agency for Medical Research and Development (AMED—19fk0210050h0001 to FT), GSK Japan Research Grant 2017 (DO), and a grant from the Nakatomi Foundation (DO).

## Conflict of Interest

The authors declare that the research was conducted in the absence of any commercial or financial relationships that could be construed as a potential conflict of interest.
