# Supplementary material for: Cellular and Mathematical Analyses of LUBAC Involvement in T Cell Receptor-Mediated NF-κB Activation Pathway
Source: Front Immunol. 2020 Nov 23;11:601926. doi: 10.3389/fimmu.2020.601926 (PMC7732508; doi:10.3389/fimmu.2020.601926)
Supplement: Supplementary file 1 [file DataSheet_1.pdf]

Supplementary Material

Cellular and Mathematical Analyses for LUBAC Involvement in T Cell Receptor-mediated NF-κB Activation Pathway

Daisuke Oikawa, Naoya Hatanaka, Takashi Suzuki, and Fuminori Tokunaga

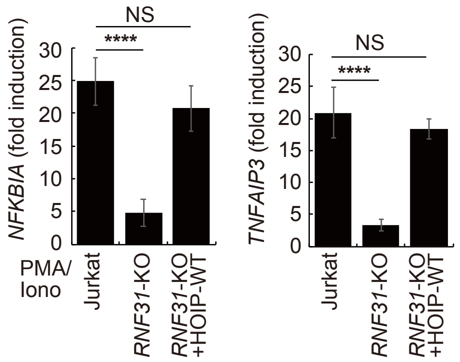

Supplementary Figure 1. Reduced expression of PMA/ionomycin-induced NF-κB target genes in *RNF31*-KO Jurkat cells. Parental, *RNF31*-KO, and WT-HOIP-restored Jurkat cells were stimulated with 20 ng/ml PMA and 150 ng/ml ionomycin for 1 h, and a qPCR analysis was performed as shown in Figure 1C. Data are Means ±SD (*n* = 3). ****: *P*<0.0001, NS: not significant.

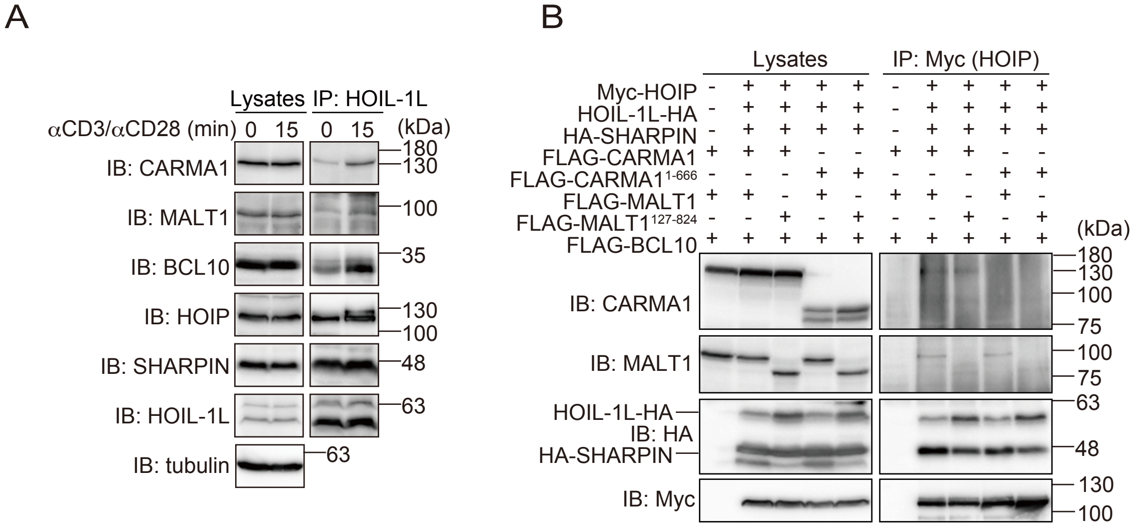

Supplementary Figure 2. Binding of LUBAC with the CBM complex. (**A**) Endogenous association of LUBAC and the CBM complex upon stimulation with anti-CD3 and anti-CD28 antibodies. Similar analysis in Figure 2A was performed after stimulation with 5 μg/ml each of anti-CD3 and anti-CD28 antibodies in Jurkat cells. The cell lysates and anti-HOIL-1L immunoprecipitates were subjected to immunoblotting with the depicted antibodies. (**B**) The effect of HOIP-binding site-deleted CARMA1 and MALT1 on binding with LUBAC. LUBAC subunits containing Myc-HOIP, HOIL-1L-HA, and HA-SHARPIN, and FLAG-CBM components were co-expressed in HEK293T cells as indicated. The cell lysates and anti-Myc immunoprecipitates were immunoblotted with the indicated antibodies.

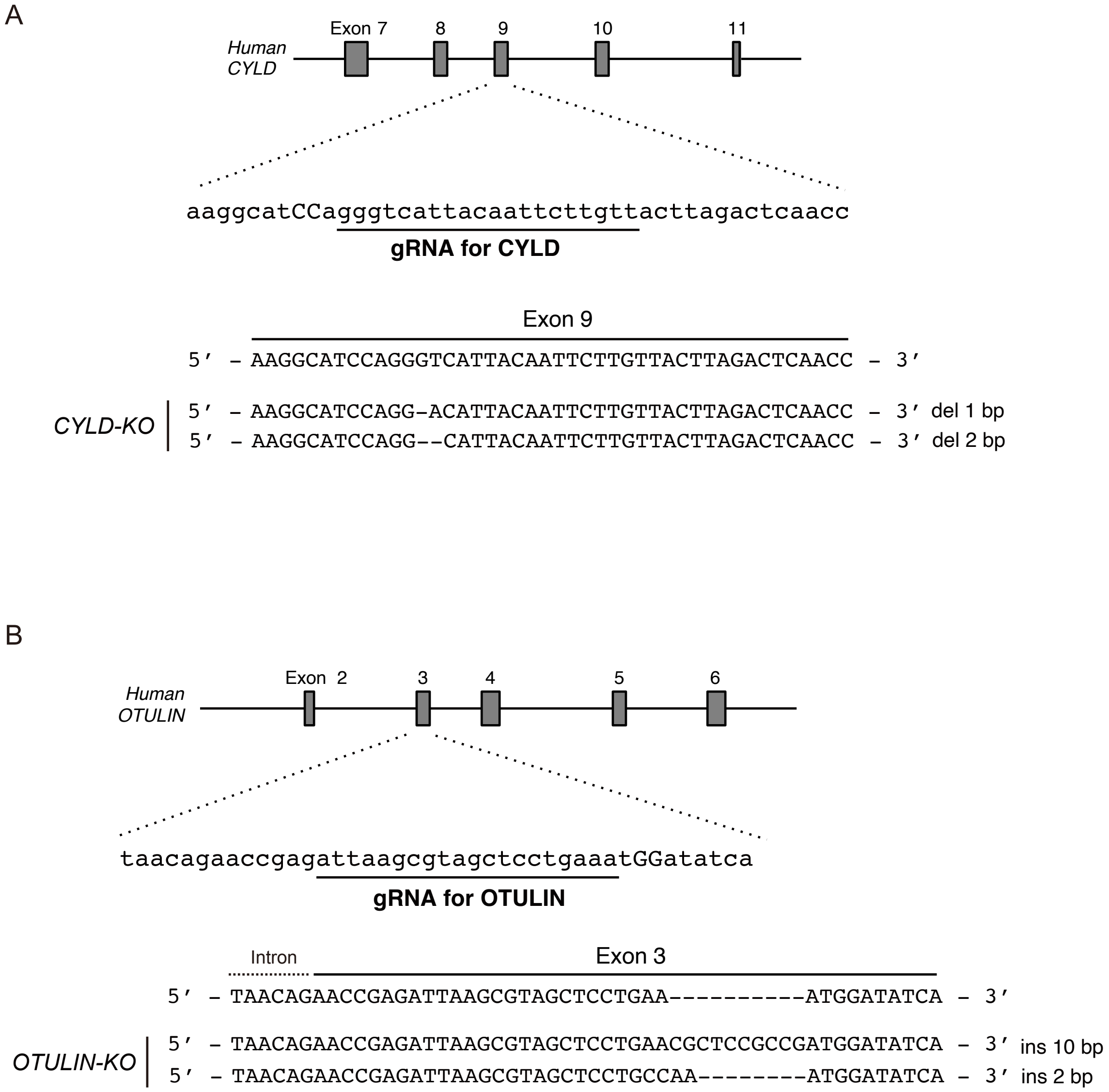

Supplementary Figure 3. Construction of *CYLD*- and *OTULIN*-deficient Jurkat cells. Schemes for *CYLD*-KO (**A**) and *OTULIN*-KO (**B**) targeting. The gRNA was employed to target exon 9 of the *CYLD* gene and exon 3 of the *OTULIN* gene. The nucleotide sequences of the constructed *CYLD*-KO and *OTULIN*-KO Jurkat cells are shown.

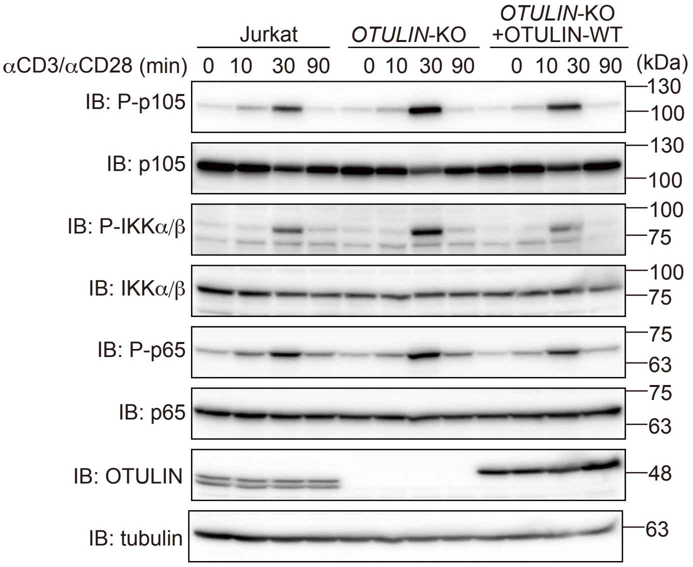

Supplementary Figure 4. Restoration of OTULIN suppressed the enhanced TCR-mediated NF-κB activation in *OTULIN*-deficient Jurkat cells. Parental, *OTULIN*-KO, and FLAG-OTULIN-WT-restored *OTULIN*-KO cells were stimulated with 3 μg/ml each of anti-CD3 and anti-CD28 antibodies for the indicated time periods, and cell lysates were immunoblotted with the depicted antibodies.
